# Recurrent pleural effusions and cardiac tamponade as possible manifestations of pseudoprogression associated with nivolumab therapy– a report of two cases

**DOI:** 10.1186/s40425-016-0185-2

**Published:** 2016-11-15

**Authors:** Bhaskar C. Kolla, Manish R. Patel

**Affiliations:** University of Minnesota, 420 Delaware St SE, MMC 480, Minneapolis, MN 55455 USA

**Keywords:** Immunotherapy, Nivolumab, Recurrent pleural effusions, Pericardial effusion, Pericardial tamponade, Lung cancer, Immune related adverse effects

## Abstract

**Background:**

Checkpoint inhibitors are a class of agents that employ host’s adaptive immune defenses in fighting cancer. With many new indications and several ongoing clinical trials in a variety of malignancies, the usage of these agents is set to increase significantly. One of the key challenges patients and physicians face while using these drugs is with the appropriate assessment of response to therapy.

**Case presentation:**

We are reporting two patients with lung cancer who were treated with nivolumab and experienced rapidly accumulating recurrent pleural effusions requiring multiple thoracenteses (6 and 4 times each for patient 1 and 2 respectively) with in the first few weeks of initiation of therapy and also developed pericardial effusion with cardiac tamponade requiring pericardiocentesis. Both patients had prior history of malignant spread to pleural and pericardial space in their disease course. Therapy was continued in the first patient with spontaneous resolution of effusions after 8 weeks and the disease showed near complete response to treatment on imaging at 16 weeks. Second patient declined to continue further treatment with nivolumab after 3 cycles due to recurrent effusions and cardiac tamponade, although there was some evidence of clinical response at discontinuation.

**Conclusions:**

Patients with history of malignant involvement of visceral spaces should be monitored closely for rapidly accumulating effusions and particularly for cardiac tamponade, after initiation of therapy with nivolumab. This presentation could represent pseudoprogression, and continuation of therapy with close monitoring is prudent as long as effusions are manageable and there is no definitive evidence of progression elsewhere.

## Background

Nivolumab is among a class of checkpoint inhibitors, which share the same mechanism of enhancing host immunity against tumor cells. Nivolumab is an IgG4 antibody that targets programmed death-1 protein (PD-1) on the T-cell surface. It acts by blocking T-cell interaction with programmed death ligand −1 protein (PDL-1) expressed by various cellular components in the tumor microenvironment [1], resulting in un-inhibition of T-cells and increased anti-tumor host immunity. As a group, checkpoint inhibitors have become a promising new addition to the cancer therapy arsenal, with some new indications and multiple ongoing clinical trials in several other malignancies.

Therefore, an increasing number of patients with cancer will be treated with this new class of drugs. One of the challenges physicians encounter with usage of these drugs is with the appropriate assessment of response to therapy [2]. It is well known that the immune-related tumor response can result in a transient increase in the size of tumors followed by regression or appearance of new lesions in presence of response to therapy elsewhere; and the response itself may take longer than that seen with traditional cytotoxic agents [3]. Biopsies of lesions have shown that transient progression followed by response (called pseudoprogression), is due to inflammation, edema and necrosis associated with immune cell infiltration of the tumor deposits [4]. Using traditional response evaluation criteria in solid tumors (RECIST) would misclassify tumor responses in such group of patients with pseudoprogression, and hence guidelines for immune-related response criteria have been proposed for evaluation of patients being treated with immunotherapy [5]. These guidelines were developed originally in melanoma patients receiving ipilimumab, and generally the incidence of pseudoprogression in solid tumor patients is thought to be low, and the way different solid tumors react to various other immunotherapy drugs may be different. In a recent review of 71 patients with metastatic non-small cell lung cancer who received anti-PD-1 therapy, only 5.6 % of patients who had treatment past progression per RECIST criteria had further tumor shrinkage [6]. Due to lack of clarity in several situations, there have been calls for increased reporting of immune related response phenomena in solid tumor patients. This should empower patients and physicians with the right knowledge when facing an important dilemma of differentiating true progression from pseudoprogression, and help them when facing crossroads of considering alternative therapies vs. continuing same treatment [7].

Here, we report two patients who developed recurrent pleural effusions and pericardial effusion with cardiac tamponade within few weeks after initiation of therapy with nivolumab. In retrospect, we postulate the likely cause could have been due to pseudoprogression.

## Case presentation 1

A 46-year old male non-smoker presented in December of 2007 with right supraclavicular lymphadenopathy. An excision biopsy of the lymph node found small cell lung cancer. A combined PET-CT (Positron Emission Tomography-Computed Tomography) scan showed a 5 cm right hilar mass and right paratracheal lymphadenopathy. He had no disease elsewhere. An MRI (Magnetic Resonance Imaging) of the brain was negative for metastatic disease.

**Fig. 1:**
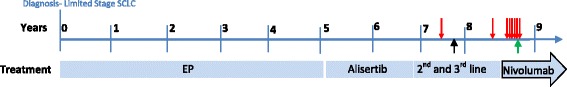
Disease course timeline for patient 1. -Pleural effusion requiring thoracentesis. -Pericardiocentesis for pericardial tamponade. -First noted to have pericardial effusion on imaging. SCLC – Small Cell Lung Cancer, EP – period during which disease was controlled using several cycles of Etoposide + Platinum, 2nd and 3rd line – period during which several second and third line agents were used including topotecan, everolimus, temozolamide, docetaxel and sunitinib

The patient was referred to our institution for treatment in January 2008. He had a low-grade disease and favorable response to various therapies, and a prolonged disease course as delineated in Fig. 1. He was initially treated with cisplatin and etoposide and concurrent radiation therapy. He achieved complete response after 6 cycles of chemotherapy, and subsequently underwent prophylactic cranial irradiation. He was monitored clinically and by imaging every 3 months. In May 2009 the disease relapsed with left supraclavicular lymphadenopathy, confirmed by excision biopsy. He underwent radiation therapy with concurrent cisplatin and etoposide for 2 cycles followed by 4 cycles of oral topotecan. He had complete response again that lasted for a year. He had 2 further courses with platinum and etoposide due to relapsed disease in 2010 and 2011. Due to relapse in his right hilar and paratracheal lymph nodes, he was treated on a phase I trial of an Aurora kinase inhibitor in 2012 with a complete response that lasted about 18 months. Then he progressed to develop aortocaval lymphadenopathy. He again received carboplatin/etoposide with initial response but developed a malignant pleural effusion and worsening retroperitoneal adenopathy after receiving 5 cycles. Over the next 18 months, he received several agents (topotecan, everolimus, temozolamide, docetaxel and sunitinib) with only stable disease as best response. His disease progressed to involve several organs including brain, spinal cord, liver, pancreas, adrenals, bone and pleural, pericardial and peritoneal spaces. During this time he underwent several palliative procedures including two resections of intramedullary metastases, multiple sessions of stereotactic brain radiation therapy, and ureteral stents to relieve obstruction.

**Fig. 2:**
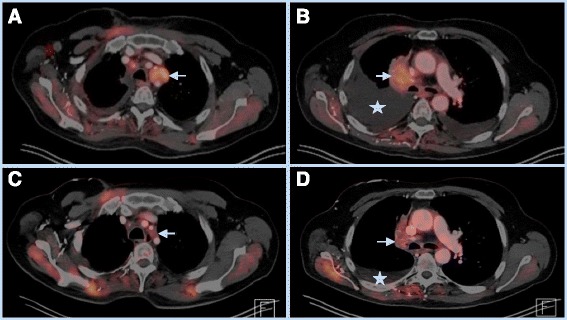
*Top*- PET-CT images July 2015 showing 3.2 cm left para-tracheal mass with SUV 6.8 (**a**) and 4.6 × 3.1 cm right para-tracheal mass with SUV 6.5 (**b**). Also seen are large right and small left pleural effusions. *Bottom*- PET-CT images from December 2015 showing complete resolution of left para-tracheal mass, and decreased size of right para-tracheal mass with equivocal hypermetabolism (SUV 2.6) along with complete resolution of left pleural effusion and residual small right pleural effusion

He was then started on nivolumab (3 mg/kg every 2 weeks) in August 2015 based on preliminary results from a phase I/II study [8]. He had a transient increase in right paratracheal tumor size causing Superior Vena Cava (SVC) syndrome that required stenting of the SVC. He also developed rapidly accumulating bilateral pleural effusions requiring a total of six thoracenteses over the next 8 weeks. He further experienced pericardial effusion with tamponade requiring pericardiocentesis on week 9 after initiation of nivolumab (Fig. 1). Cytologies from both pleural and pericardial fluid were positive for malignancy. Pericardial fluid cytology showed 5 % lymphocytes. The treatment was continued every 2 weeks without any break. He had evidence of partial response at 8 weeks of therapy and near complete response at 16 weeks of therapy in December 2015 (Fig. 2). He did not require any further pleural or pericardial drainage after the first 2 months of therapy, and he continues to remain on treatment to date.

## Case presentation 2

Patient 2 is 54-year-old female non-smoker, who was diagnosed with adenocarcinoma of lung with liver metastases in May 2012. Molecular analysis revealed EGFR (Epidermal Growth Factor Receptor) mutation on exon 21. She was mostly treated at a different institution and visited us a few times for second opinion. She was initially started on erlotinib and she went to her home country where it was switched to gefitinib due to skin rash. She had marked improvement on follow-up PET scan. She underwent stereotactic body radiation therapy (SBRT) to the primary lung lesion in right lower lobe and to the hepatic metastases. She returned to United States in March 2013 when therapy was switched back to erlotinib without any side effects. In June 2013 she developed pleural effusion on the right side and underwent thoracenteses. Cytology was positive for malignancy. She decided to bring gefitinib from Taiwan and started using it due to progression. In November 2013, she developed pericardial effusion with tamponade and underwent pericardiocentesis. Therapy was switched to afatinib. She showed objective response, however in April 2014 she developed progression with metastases to uterus. She was treated with bevacizumab plus afatinib. She had stable disease for about a year when she progressed in May 2015.

**Fig. 3:**
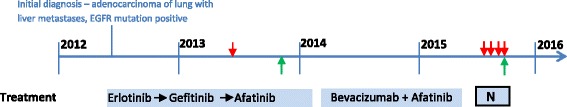
Disease course timeline for patient 2. -Pleural effusion requiring thoracentesis. -Pericardial tamponade requiring pericardiocentesis. N- Nivolumab

In July 2015, she was started on nivolumab (3 mg/kg every 2 weeks). She developed recurrent right pleural effusions requiring four thoracenteses over the next 8 weeks (Fig. 3). She also developed pericardial effusion with cardiac tamponade and underwent pericardiocentesis 7 weeks after initiation of nivolumab. Both pleural and pericardial fluid cytologies were positive for malignancy. Lymphocytes accounted for 30 % of cells in pericardial fluid analysis. She was also treated with prednisone for the possibility of immune-related Adverse Effect (irAE). Doses varied between 20– 60 mg daily due to successive tapering schedules with recurrent effusions. Although her metastatic thyroid nodule and metastatic skin nodules showed clinical response after 3 treatments, the patient declined further treatment with nivolumab after she was admitted to intensive care for 4 days due to pericardial tamponade on week 7 of therapy. Unfortunately further attempts to restart nivolumab by her providers after a good recovery were also declined by the patient. Within 3 months of discontinuation, the patient again had progressive disease and is currently on therapy with osimertinib (due to detection of EGFR T790M mutation).

## Discussion

Both of these patients had a history of malignant pleural and pericardial effusions in their disease course prior to the treatment with nivolumab. Following initiation of therapy with nivolumab, they developed recurrent pleural effusions that re-accumulated rapidly within few days after each tap, needing multiple thoracenteses with in the first 8 weeks. Both patients also developed pericardial effusion with tamponade requiring pericardiocentesis. There have been no previous reports to our knowledge in the literature that described this clinical presentation [9].

With respect to recurrent effusions in patient 1, we considered the possibilities of irAE vs. pseudoprogression initially; however, as the patient was otherwise doing well clinically, we were able to manage his recurrent effusions with repeated fluid aspirations while following him closely. In retrospect, it seems likely that the recurrent effusions may have been secondary to pseudoprogression, as they occurred within the first few weeks of therapy concurrently with tumor enlargement elsewhere, and they stopped spontaneously without recurrence after 8 weeks, concurrently with evidence of response to therapy at other places. One would have expected irAE to worsen and not resolve spontaneously with continued therapy. In case of our second patient who was treated at an outside institution at the same time as patient 1, the clinical course followed a similar pattern as seen in patient 1 with respect to recurrent effusions and pericardial tamponade. She also had an initial increase in the size of her metastatic thyroid nodule and skin metastases followed by partial regression after cycle 3. Though we discussed cautiously monitoring her without steroids, the patient decided in consultation with her primary oncologist to initiate prednisone in addition to repeated fluid aspiration, and subsequently discontinued therapy after cycle3. Because of the steroid use and the discontinuation of nivolumab, it is difficult to conclude with any certainty the mechanism of recurrent effusion in this case; however, the fact that she did show some improvement in disease elsewhere suggest that it could be due to pseudoprogression or irAE. Prior experience with similar clinical situations or a clear understanding of the mechanisms behind this clinical phenomenon would have provided a stronger argument for careful continuation of therapy with close monitoring. The variation seen in response to a similar clinical situation between different providers highlights the importance of need for increased reporting of these phenomena, and for studies to understand the underlying mechanisms, in patients undergoing immunotherapy.

In both cases, the possibility of immune-related serositis secondary to nivolumab was considered, however, there was no massive lymphocyte infiltration in the pericardial fluid analysis in either case. Lymphocytes accounted for 5 % of cells in patient 1, and 30 % of cells in patient 2. In retrospect, serial flow cytometry of pleural fluid may have been instructive to better characterize the ongoing changes within the pleural fluid over time. In both phase 3 trials of nivolumab vs. docetaxel, pleural or pericardial effusion as adverse events were not common [10, 11]. In the squamous non-small cell lung cancer trial, pleural or pericardial effusions were not reported as adverse events [11]. Of all-cause adverse events in the non-squamous non-small cell lung cancer trial, pleural effusion was reported in 6 % of patients in the nivolumab arm and 3 % of patients in the docetaxel arm. Of the treatment-related serious adverse events, pleural effusion was not reported and pericardial effusion was reported in 1 out of 287 patients (<1 %) in the nivolumab arm [10]. No attribution of cause is described in the manuscript for the 6 % of patients who developed pleural effusions in the nivolumab arm; however, we would presume that most of these would be attributable to progressive disease. It is furthermore unknown, how many patients had pre-existing pleural or pericardial effusions prior to the initiation of nivolumab. Therefore, the above cases highlight the need to more carefully evaluate the clinical scenario in patients with worsening effusions, especially early after initiation of nivolumab therapy.

## Conclusion

In conclusion, patients with history of malignant pleural or pericardial effusions should be monitored closely for recurrent effusions after initiation of nivolumab therapy. Such presentation could represent pseudoprogression and possibly a harbinger of response to therapy. We would posit that careful continuation of nivolumab without initiation of steroids may be the best approach as long as the effusions can be managed with drainage, unless there is clear evidence of progression elsewhere. Careful analysis of fluid with flow cytometry should be considered in such cases as a large increase in lymphocytes may be an indication for initiation of steroids. Increased reporting of these immune related phenomena and studies to understand mechanisms behind such presentation, are necessary to guide patients and physicians with appropriate course of action.

## Abbreviations

EGFR: Epidermal growth factor receptor
IrAE: Immune-related adverse effect
MRI: Magnetic resonance imaging
PD-1: Programmed death −1
PDL-1: Programmed death ligand – 1
PET-CT: Positron emission tomography – computed tomography
RECIST: Response evaluation criteria in solid tumors
SVC: Superior vena cava
